# Yb-Doped BaCeO_3_ and Its Composite Electrolyte for Intermediate-Temperature Solid Oxide Fuel Cells

**DOI:** 10.3390/ma12050739

**Published:** 2019-03-04

**Authors:** Xueyue Jiang, Fufang Wu, Hongtao Wang

**Affiliations:** School of Chemical and Material Engineering, Fuyang Normal College, Anhui Provincial Key Laboratory for Degradation and Monitoring of Pollution of the Environment, Fuyang 236037, China; jiangxueyue@126.com

**Keywords:** defects, composite, electrolytes, hydrogen, fuel cell, conductivity

## Abstract

BaCe_0.9_Yb_0.1_O_3−α_ was prepared via the sol-gel method using zirconium nitrate, ytterbium trioxide, cerium nitrate and barium acetate as raw materials. Subsequently, it reacted with the binary NaCl~KCl salt to obtain BaCe_0.9_Yb_0.1_O_3−α_-NaCl~KCl composite electrolyte. The structure, morphology, conductivity and fuel cell performance of the obtained samples were investigated. Scanning electron microscope (SEM) images showed that BaCe_0.9_Yb_0.1_O_3−α_ and NaCl~KCl combined with each other to form a homogeneous 3-D reticulated structure. The highest power density and conductivity of BaCe_0.9_Yb_0.1_O_3−α_-NaCl~KCl was 393 mW·cm^−2^ and 3.0 × 10^−1^ S·cm^−1^ at 700 °C, respectively.

## 1. Introduction

Fuel cells have many merits, such as diversity of fuel options, being environmentally friendly and having high energy efficiency [1,2,3,4,5,6,7,8]. BaCeO_3_ and SrCeO_3_-based perovskite oxides have excellent protonic conductivities under hydrogen- or water-containing atmosphere at 400–1000 °C [9,10,11,12,13,14,15]. The oxygen vacancies appear when Ce^4+^ is substituted with trivalent metal cations [16]. Owing to the concentrations of oxygen vacancies and point defect pairs, two opposing factors, the optimum doping level of BaCeO_3_ and SrCeO_3_-based electrolytes is usually 10% [17]. Among these doped metal cations, Y^3+^ and Yb^3+^ doped BaCeO_3_ or SrCeO_3_ have relatively high conductivities [17,18]. The synthetic methods of BaCeO_3_ and SrCeO_3_-based electrolytes are solid-state reactions, citrate-nitrate combustions, microemulsions and sol-gel methods [19,20]. The solid-state reaction method requires a high temperature (1550–1700 °C) and the particle size of the product is larger. By comparison, the sol-gel method can mix raw materials at the nanometre level. Moreover, the sintering temperature can be reduced to 200–300 °C.

Intermediate temperature solid oxide fuel cells have many advantages, such as good selectivity, durability and low cost [21,22,23,24]. The excellent protonic conduction of BaCeO_3_-based electrolytes is mainly reflected at high temperatures (700–1000 °C). Also, the conductivities of BaCeO_3_-based electrolytes are relatively low at intermediate temperatures (400–700 °C). In applying BaCeO_3_-based electrolytes to intermediate temperature solid oxide fuel cells, electrolyte membranes and composite electrolytes have attracted intensive attention in recent years [25,26,27,28,29,30,31,32]. Park et al. reported that the conductivities of composite BaZr_0.85_Y_0.15_O_3−δ_-Nd_0.1_Ce_0.9_O_2−δ_ electrolyte are higher than that of single BaZr_0.85_Y_0.15_O_3−δ_ above 600 °C [28]. Huang et al. found the conductivities of BaCe_0.7_Zr_0.1_Y_0.2_O_3−δ_-Li_2_CO_3_-Na_2_CO_3_ composite electrolyte >0.1 S·cm^−1^ at 600 °C [32]. Our previous studies indicated that SrCeO_3_-based oxides-inorganic salt composite electrolytes have good intermediate temperature electrochemical properties [33,34]. Usually, BaCeO_3_-based electrolytes have higher conductivities than SrCeO_3_-based ones. To date, there are only a few reports on composite electrolytes of BaCeO_3_-based ceramic/carbonate [32]. BaCeO_3_-based electrolytes/chloride composite electrolytes have not been developed and investigated thoroughly.

In this study, BaCe_0.9_Yb_0.1_O_3−α_ was prepared via the sol-gel method and the composite electrolyte of BaCe_0.9_Yb_0.1_O_3−α_-NaCl~KCl was also synthesized. The morphology, physical chemistry change, and the structure of BaCe_0.9_Yb_0.1_O_3−α_ were studied using SEM, Thermogravimetric Analysis and Differential Scanning Calorimetry (TGA-DSC) and X–ray diffractometer (XRD). The intermediate temperature electrochemical properties of BaCe_0.9_Yb_0.1_O_3−α_ and BaCe_0.9_Yb_0.1_O_3−α_-NaCl~KCl were also investigated.

## 2. Materials and Methods

BaCe_0.9_Yb_0.1_O_3−α_ was prepared via the sol-gel method using zirconium nitrate, ytterbium trioxide, cerium nitrate and barium acetate as the raw materials. The stoichiometric metal ion salts (Ba^2+^:Ce^4+^:Yb^3+^ = 10:9:1) were dissolved in deionized water. Citric acid was added (three times as much as the metal ion salts). The *p*H of the above solution was adjusted to 8.0 with ammonia and heated at 90 °C for 6 h until gelatinous. The xerogel was obtained at 130 °C and heated for the ashing treatment [35,36,37]. The calcination of the resultant ash was carried out at 1250 °C and 1550 °C for 5 h, respectively, to obtain BaCe_0.9_Yb_0.1_O_3−α_.

A 1:1 mole ratio of NaCl to KCl was heated at 700 °C to form the molten salt [38]. The weight ratio of BaCe_0.9_Yb_0.1_O_3−α_:NaCl~KCl = 80:20 was mixed and ground. Then, the mixing powders were sintered at 750 °C for 2 h to obtain BaCe_0.9_Yb_0.1_O_3−α_-NaCl~KCl.

Thermogravimetric Analysis and Differential Scanning Calorimetry (TGA-DSC, Universal V 3.7A, TA Instruments, New Castle, DE, USA) were conducted before and after the ashing treatment of the BaCe_0.9_Yb_0.1_O_3−α_ precursor. The temperature ranged between 25 °C and 1100 °C with a heating rate of 15 °C·min^−1^. The structures of BaCe_0.9_Yb_0.1_O_3−α_ (1250 °C, 1550 °C) and BaCe_0.9_Yb_0.1_O_3−α_-NaCl~KCl were determined by X–ray diffractometer (XRD, X’pert Pro MPD, Holland’s company, Amsterdam, Netherlands). From the X-ray spectrogram, the average crystallite size (D_XRD_) can be calculated from:

D_XRD_ = 0.89λ/bcos θ (1)

where λ is the X-ray wavelength of Cu-Kα radiation (λ = 0.15405 nm), b is the corrected half-width of the diffraction peak and θ is the diffraction angle (°) [35]. The external and cross-sectional surfaces of BaCe_0.9_Yb_0.1_O_3−α_ (1550 °C) and BaCe_0.9_Yb_0.1_O_3−α_-NaCl~KCl were imaged using a scanning electron microscope (SEM, S-4700, Hitachi, Tokyo, Japan).

For conductivity measurements, BaCe_0.9_Yb_0.1_O_3−α_ (1550 °C) and BaCe_0.9_Yb_0.1_O_3−α_-NaCl~KCl pellets were processed into wafers (diameter = 16 mm, thickness = 1.0 mm). The electrodes (area = 0.50 cm^2^) were comprised of 20 wt% Pd and 80 wt% Ag and the wires were pure Ag. The conductivities were investigated utilizing an electrochemical analyzer over the frequency range from 1 Hz to 100 KHz in the air at 400–700 °C as well as with the oxygen partial pressures (*p*O_2_) from 1 × 10^−20^ to 1 atm at 700 °C [8]. The electrochemical impedance spectroscopy (EIS) of BaCe_0.9_Yb_0.1_O_3−α_ (1550 °C) and BaCe_0.9_Yb_0.1_O_3−α_-NaCl~KCl were studied under open circuit conditions. Finally, H_2_/O_2_ fuel cells were fabricated and tested.

## 3. Results and Discussion

**Figure 1:**
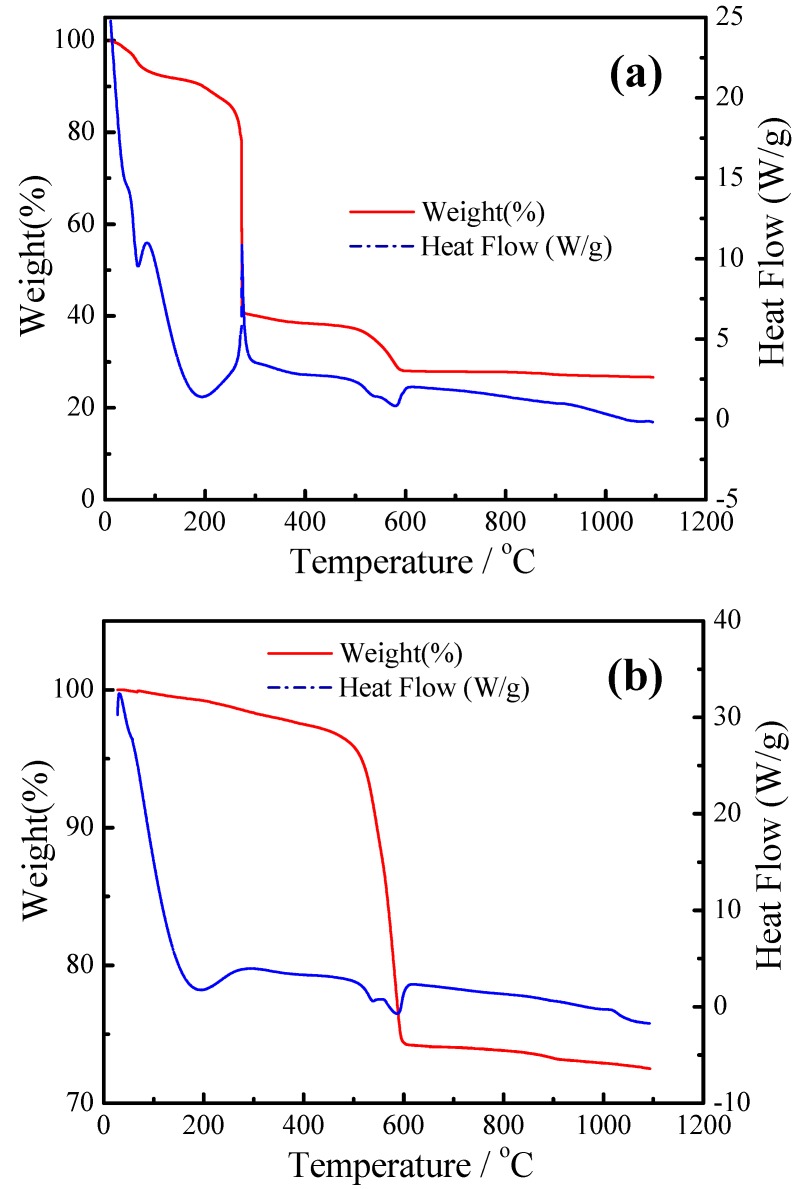
Thermogravimetric Analysis and Differential Scanning Calorimetry (TGA-DSC) plots for the BaCe_0.9_Yb_0.1_O_3−α_ precursor before (**a**) and after (**b**) ashing treatment.

TGA-DSC plots for the BaCe_0.9_Yb_0.1_O_3−α_ precursor were measured before and after the ashing treatment. In Figure 1a, the DSC curve has a sharp exothermic peak between 260 °C and 300 °C accompanied by 45% weight loss, mainly attributed to the decomposition of citric acid and ammonium salt. The weight loss is gentle, declining from 510 °C to 580 °C, which is attributed to the decomposition of the nitrate. As seen in Figure 1b, there was a decline in weight loss around 550 °C, which is ascribed to the incomplete decomposition of the nitrate [39,40]. There was almost no weight loss after 1070 °C indicating that the BaCeO_3_ phase had begun to form.

**Figure 2:**
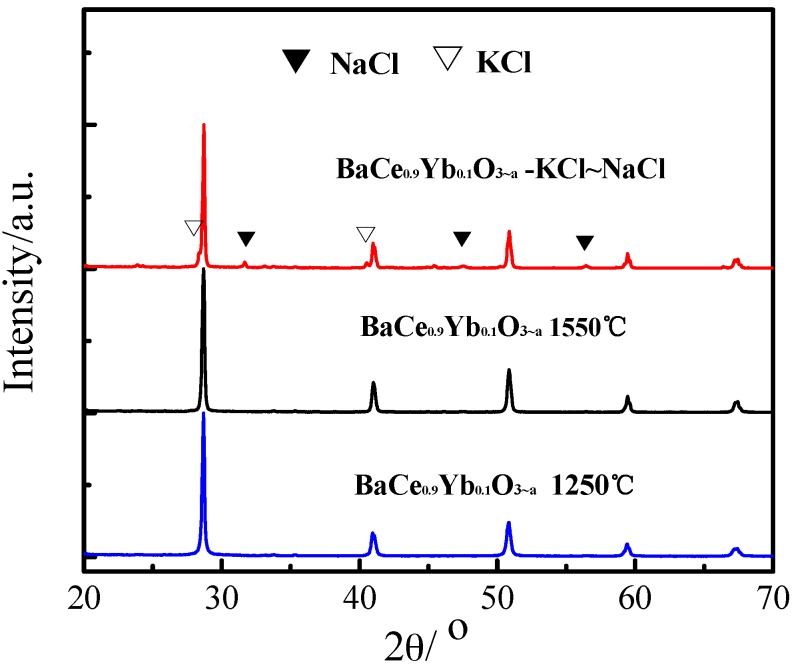
X–ray diffractometer (XRD) patterns of BaCe_0.9_Yb_0.1_O_3−α_ (1250 °C, 1550 °C) and BaCe_0.9_Yb_0.1_O_3−α_-NaCl~KCl.

The XRD patterns of BaCe_0.9_Yb_0.1_O_3−α_ (1250 °C, 1550 °C) and BaCe_0.9_Yb_0.1_O_3−α_-NaCl~KCl are shown in Figure 2. The XRD patterns show that the sintered BaCe_0.9_Yb_0.1_O_3−α_ (1250 °C, 1550 °C) samples are all orthorhombic BaCeO_3_ phases. The average crystallite sizes (D_XRD_) of BaCe_0.9_Yb_0.1_O_3−α_ (1250 °C, 1550 °C) samples are 45.9573 nm and 50.2176 nm, respectively. Combined with the results of Figure 1, the first sintering temperature of 1250 °C is suitable. There are some small additional peaks in the BaCe_0.9_Yb_0.1_O_3−α_-NaCl~KCl XRD spectrum, suggesting that NaCl~KCl inorganic salts exist as crystalline phases in the composite electrolyte [35].

**Figure 3:**
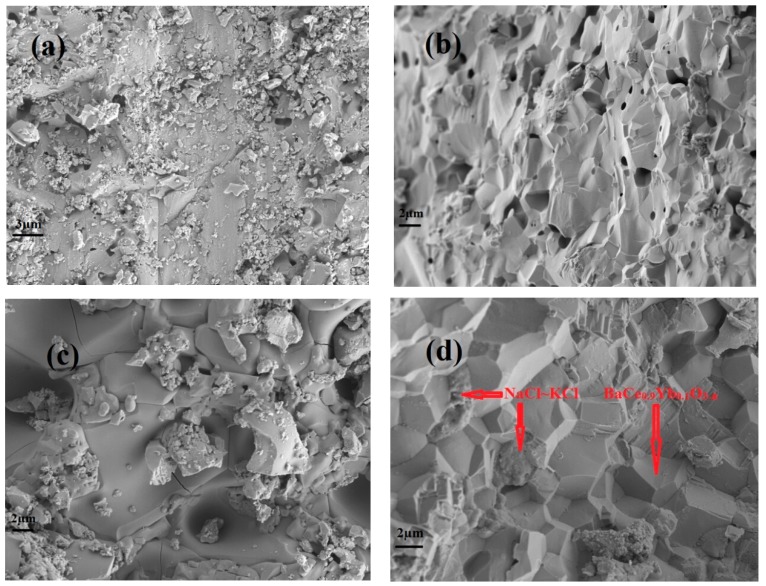
Scanning electron microscope (SEM) photos of BaCe_0.9_Yb_0.1_O_3−α_ calcined at 1550 °C for 5 h (**a**,**b**) external and cross-sectional surfaces, and BaCe_0.9_Yb_0.1_O_3−α_-NaCl~KCl sintered at 750 °C for 2 h (**c**,**d**) external and cross-sectional surfaces.

The SEM external and cross-sectional surface images of BaCe_0.9_Yb_0.1_O_3−α_ calcined at 1550 °C for 5 h (Figure 3a,b) and BaCe_0.9_Yb_0.1_O_3−α_-NaCl~KCl sintered at 750 °C for 2 h (Figure 3c,d) are displayed in Figure 3. As seen in Figure 3a,b, the degree of BaCe_0.9_Yb_0.1_O_3−α_ particle agglomeration is good. However, the fractured surface image of BaCe_0.9_Yb_0.1_O_3−α_ shows that there are still some holes after being calcined at 1550 °C for 5 h, as shown in Figure 3b. It has been proved by our experiments that they are closed holes. In Figure 3c,d, it is clearly visible that the particles of BaCe_0.9_Yb_0.1_O_3−α_ are aggregated into clumps after the addition of NaCl~KCl inorganic salts sintered at 750 °C for 2 h. The regular polyhedron zones correspond to the BaCe_0.9_Yb_0.1_O_3−α_. Contrastingly, the amorphous areas point to the NaCl~KCl inorganic salt phase. Combined with the results of Figure 2, NaCl~KCl inorganic salts exist as both crystalline and amorphous phases [31,32].

**Figure 4:**
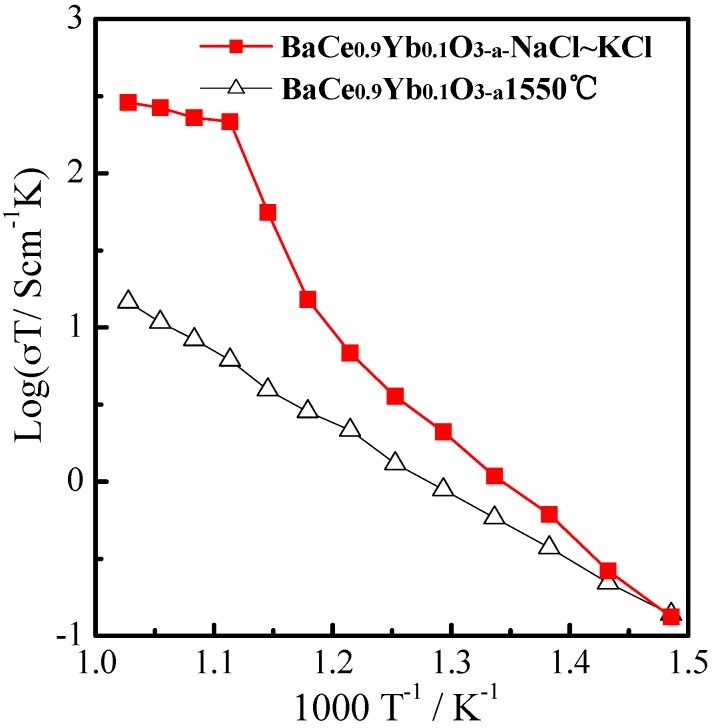
The log (σT)~1000 T^−1^ plots of BaCe_0.9_Yb_0.1_O_3−α_ (1550 °C) and BaCe_0.9_Yb_0.1_O_3−α_-NaCl~KCl in the air from 400 °C to 700 °C.

Figure 4 shows the log (σT)~1000 T^−1^ plots of BaCe_0.9_Yb_0.1_O_3−α_ (1550 °C) and BaCe_0.9_Yb_0.1_O_3−α_-NaCl~KCl in the air from 400 °C to 700 °C. As seen in Figure 4, the conductivities of composite BaCe_0.9_Yb_0.1_O_3−α_-NaCl~KCl electrolytes are higher than that of the single BaCe_0.9_Yb_0.1_O_3−α_. The conductivities of BaCe_0.9_Yb_0.1_O_3−α_-NaCl~KCl vary from 2.0 × 10^−4^ S·cm^−1^ to 3.0 × 10^−1^ S·cm^−1^ in the range of 400–700 °C which is equivalent to BaZr_0.85_Y_0.15_O_3−α_-Li_2_CO_3_-K_2_CO_3_ in the air at 650 °C [31]. The single BaCe_0.9_Yb_0.1_O_3−α_ electrolyte shows a linear Arrhenius curve in the air at 400–700 °C, whereas the conductivities of BaZr_0.85_Y_0.15_O_3−α_-Li_2_CO_3_-K_2_CO_3_ start to increase dramatically above 600 °C. The results indicate that the molten NaCl~KCl salt provides more ion transport channels at high temperatures [31,32,41].

**Figure 5:**
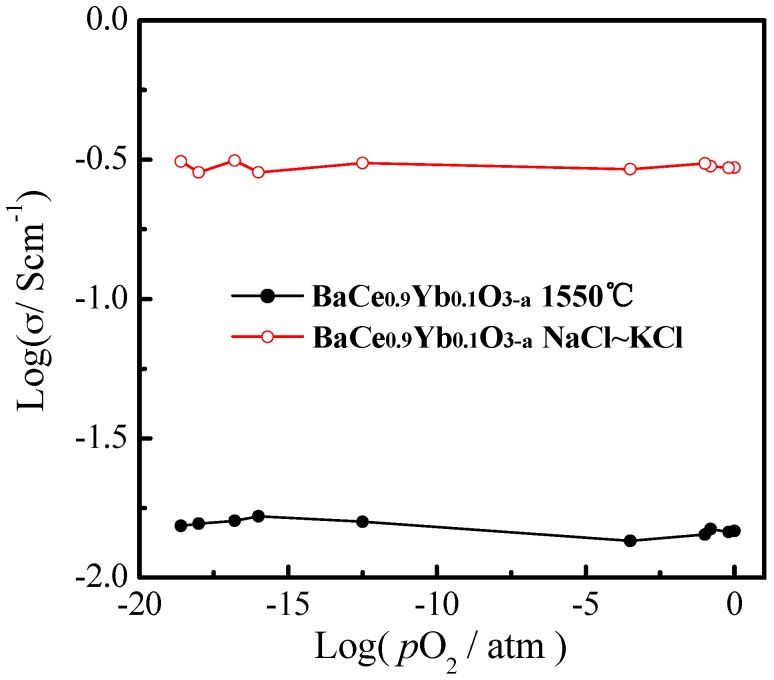
The conductivities of BaCe_0.9_Yb_0.1_O_3−α_ (1550 °C) and BaCe_0.9_Yb_0.1_O_3−α_-NaCl~KCl as a function of *p*O_2_ at 700 °C.

Figure 5 shows the conductivities of BaCe_0.9_Yb_0.1_O_3−α_ (1550 °C) and BaCe_0.9_Yb_0.1_O_3−α_-NaCl~KCl as a function of *p*O_2_ from 1 × 10^−20^ to 1 atm at 700 °C. The log *σ* ~ log *p*O_2_ plot is usually used to estimate the ionic and electronic conduction of an electrolyte. Pikalova et al. reported that BaCe_0.89_Gd_0.1_Cu_0.01_O_3−α_ has a predominantly proton-conducting character at intermediate and low *p*O_2_ values [9]. As shown in Figure 5, the conductivity is a horizontal line parallel to the *X*-axis, which indicates that the samples are almost pure ionic conductors. This may be ascribed to the molten salts acting as fast conduction paths for ionic charge carriers, which corresponds with related reports on composite electrolytes [25,26,27,28,29,30,31,32].

**Figure 6:**
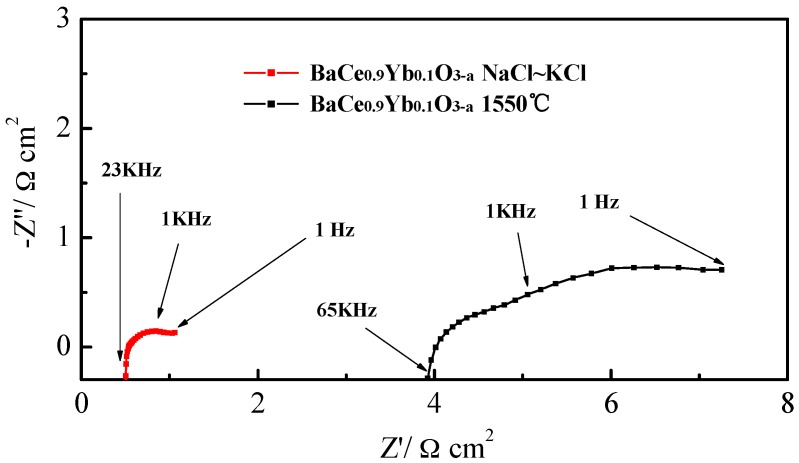
The electrochemical impedance spectroscopy (EIS) of BaCe_0.9_Yb_0.1_O_3−α_ (1550 °C) and BaCe_0.9_Yb_0.1_O_3−α_-NaCl~KCl under open-circuit conditions at 700 °C.

Figure 6 presents the electrochemical impedance spectroscopy (EIS) of BaCe_0.9_Yb_0.1_O_3−α_ (1550 °C) and BaCe_0.9_Yb_0.1_O_3−α_-NaCl~KCl under open-circuit conditions at 700 °C. Usually, the AC impedance curve includes a semicircle and a radial at high (1 KHz–100 KHz) and low (1 Hz–1 KHz) frequencies which correspond to the ohmic and total resistances, respectively. Additionally, the difference between them from the intercept with the real axis at high frequencies to the juncture point of the semicircle and radial, represents polarization resistance (*R*_p_) [18]. The semicircle gradually disappears as the temperature increases [42,43]. In Figure 6, the polarization resistance (*R*_p_) for BaCe_0.9_Yb_0.1_O_3−α_ (1550 °C) and BaCe_0.9_Yb_0.1_O_3−α_-NaCl~KCl are 1.72 Ω·cm^2^ and 0.31 Ω·cm^2^, respectively. This result indicates that the molten salt cannot only generate fast transport ways but also enhance its long-range mobility, which leads to lower resistance and higher performance.

**Figure 7:**
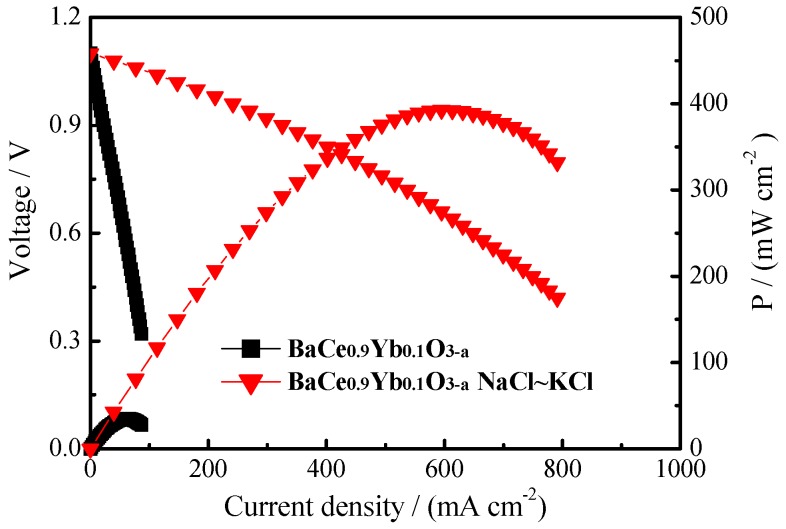
The *I*–*V*–*P* curves of BaCe_0.9_Yb_0.1_O_3−α_ (1550 °C) and BaCe_0.9_Yb_0.1_O_3−α_-NaCl~KCl at 700 °C.

Figure 7 shows the *I*–*V*–*P* curves of BaCe_0.9_Yb_0.1_O_3−α_ (1550 °C) and BaCe_0.9_Yb_0.1_O_3−α_-NaCl~KCl at 700 °C. The following reactions occur in the cathode and anode compartments:

cathode reaction: 2H^+^ + O_2_ + 4e^−^ = H_2_O + O^2−^ (2)

and

anode reaction: 2H_2_ + O^2−^ = 2H^+^ + H_2_O + 4e^−^. (3)

The H_2_/O_2_ fuel cell using BaCe_0.9_Yb_0.1_O_3−α_-NaCl~KCl (thickness = 1.0 mm) as electrolyte achieves the highest power density (*P*_h_) of 393 mW·cm^−2^ when the voltage is 0.64 V at 700 °C. The SrCe_0.6_Zr_0.3_Lu_0.1_O_3−α_ only has 34.8 mW·cm^−2^ under the same conditions. The *P*_h_ value of our result is higher than the fuel cell performance of 60 wt% Ce_0.8_Sm_0.2_O_1.9_-40 wt% (Li/Na)_2_CO_3_ (575 °C) and BaCe_0.7_In_0.15_Ta_0.05_Y_0.1_O_3−δ_ (thickness = 25 µm, 700 °C), however, lower than 80 wt% BaCe_0.7_Zr_0.1_Y_0.2_O_3−δ_-20 wt% (Li/Na)_2_CO_3_ (thickness = 0.4 mm, 600 °C) as shown in Table 1 [18,32,44]. This may be due to the different electrolyte and inorganic salt types and fuel cell construction.

## 4. Conclusions

In this study, BaCe_0.9_Yb_0.1_O_3−α_ was prepared via the sol-gel method. The first sintering temperature for the BaCe_0.9_Yb_0.1_O_3−α_ precursor was determined using TGA-DSC. XRD and SEM results indicated that NaCl~KCl inorganic salts exist as both crystalline and amorphous phases. The polarization resistances (*R*_p_) for BaCe_0.9_Yb_0.1_O_3−α_ (1550 °C) and BaCe_0.9_Yb_0.1_O_3−α_-NaCl~KCl were 1.72 Ω·cm^2^ and 0.31 Ω·cm^2^ under open-circuit conditions at 700 °C, respectively. The highest power density and conductivity of BaCe_0.9_Yb_0.1_O_3−α_-NaCl~KCl were 393 mW·cm^−2^ and 3.0 × 10^−1^ S·cm^−1^ at 700 °C, respectively.

## Figures and Tables

**Table 1:** The highest power densities of BaCe_0.9_Yb_0.1_O_3−α_-NaCl~KCl and similar electrolytes in the literature.

Electrolytes	Highest Power Densities
BaCe_0.9_Yb_0.1_O_3−α_-NaCl~KCl (80: 20)	393 mW·cm^−2^ (thickness = 1.0 mm), 700 °C, in this work
BaCe_0.7_Zr_0.1_Y_0.2_O_3−δ_-(Li/Na)_2_CO_3_ (80: 20)	957 mW·cm^−2^ (thickness = 0.4 mm), 600 °C, [32]
Ce_0.8_Sm_0.2_O_1.9_-(Li/Na)_2_CO_3_ (60: 40)	240 mW·cm^−2^, 575 °C, [44]
BaCe_0.7_In_0.15_Ta_0.05_Y_0.1_O_3−δ_	303 mW·cm^−2^ (thickness = 25 µm), 700 °C, [18]
